# Evaluating an Innovative Oncology Education Initiative: Integrating Genomics and Histopathology in a Virtual Laboratory e-Learning Resource to Enhance Learner Engagement

**DOI:** 10.1177/23821205251406438

**Published:** 2026-01-24

**Authors:** Karen R. Reed, Owen J. Crawford, Hannah D. West, Joseph Lewis, Michael Hackman, Andreia de Almeida, Andrew Hilbourne, Emma Short, Sarju Patel, Keith Hart, Athanasios Hassoulas

**Affiliations:** 1 Centre for Medical Education, 2112 School of Medicine, Cardiff University, Cardiff, UK; 2 Cardiff Learning & Teaching Academy, 2112 Cardiff University, Cardiff, UK; 3 Department of Cellular Pathology, 574412 University Hospital of Wales, Cardiff, UK; 4 7757 Cellular Pathology, Swansea Bay University Health Board, Morriston Hospital, Swansea, UK

**Keywords:** virtual reality, VR, genomics, histopathology, oncology, technology-Enhanced learning

## Abstract

Advances in genomics have transformed histopathology, especially in oncology teaching. Histopathologists traditionally assess tissue morphology, but now integrate genomic data to enhance diagnostic accuracy and treatment precision. Students must grasp both disciplines and appreciate the multi-disciplinary nature of modern oncology. To address this, and deliver content in an engaging way, we developed a computer-based virtual reality laboratory experience (the VR-Lab), seamlessly integrating genomics and histopathology into case-based learning, and undertook descriptive evaluation of user engagement with the VR-Lab. This demonstrated enhanced student engagement compared to a conventional workbook, although the provision of a complementary workbook was beneficial for some. We conclude that integrating technology-enhanced learning approaches, such as the VR-Lab, alongside traditional resources like workbooks can enhance student engagement with teaching materials and provide students with the options to choose an approach that suits their learning preferences.

## Introduction

Advances in genomics have significantly impacted the field of histopathology, particularly in the context of oncology teaching. Histopathology, which involves the examination of tissue samples under a microscope, plays a crucial role in diagnosing and understanding diseases, in providing prognostic information and in guiding therapies. Simultaneously, genomics (the study of our genes and their functions) has revolutionised our understanding of cancer and personalised treatment options. While histopathologists have traditionally assessed tissue morphology, including tissue architecture and cellular changes associated with various diseases, their role has evolved. In addition to microscopic assessment, histopathologists often integrate genomic data with morphological information to inform clinical decisions. Understanding genetic alterations in the context of the patient's clinical history and tissue morphology, enhances diagnostic accuracy and treatment precision.^1–3^

As genomics continues to advance, students and practitioners alike must embrace this interdisciplinary approach to improve patient outcomes. However, a challenge medical educators face is ensuring students have a solid understanding of the principles of both disciplines, while recognising that students often consider both to be challenging topics.^4,5^ Furthermore, beyond the need to grasp the biological/scientific aspects required to bridge the gap between genomics and histopathology, students must understand that modern oncology is multi-disciplinary and requires collaboration among: pathologists; clinicians; radiologists; clinical nurse specialists; geneticists; and bioinformaticians. Understanding this helps students grasp the practical implications and therapeutic strategies in clinical practice.

Here, we describe the development and initial evaluation of a computer-based virtual reality laboratory experience (the VR-Lab), which aimed to integrate genomics and histopathology seamlessly in an engaging way to aid student learning. This was achieved by utilising a narrative and case-based learning approach to provide clear explanations and enhance students’ comprehension of these critical topics.^6–8^ VR technologies and other tools that promote greater interactivity with the learning content, are not only popular with students but, have been shown to be effective tools to aid learning.^9–11^ Indeed, a study evaluating teaching methods utilising technology integration developed during the 2020 COVID-19 pandemic, demonstrated that student engagement in histology teaching content delivered using e-learning platforms and virtual tools was improved, without adversely affecting student learning.^12^ Added to this, many studies have demonstrated that improved engagement leads to improved learning and improvements in attainment.^13–15^ Thus, we believe that the use of such technology-enhanced approaches would improve student engagement and learning.

In previous years, the taught content had been delivered using a flipped classroom approach,^16,17^ using an online workbook (in the form of a word document) to deliver the content, followed by an in-person question-and-answer (Q&A) session. While this approach had received positive student feedback, we believed that using technology-enhanced learning approaches, such as employing VR technology, could improve content delivery to aid student engagement and subsequent learning.^18^ Thus, we aimed to utilise this technology to provide content in an engaging way, in which students could move around the VR-lab and actively engage with the technology and tools used in each process in order to show how genomic alterations manifest at the tissue level, illustrating the interplay between genomics and histopathology, and presenting a narrative that was able to connect theoretical knowledge to a simulated patient case. We chose to maintain the flipped learning approach and provided students the choice of using either the conventional workbook, the VR-Lab, or both, as tools to engage with the core content. Evaluation conducted after the Q&A session showed we met our aims.

## Methods

### Resource Development

**Figure 1.**
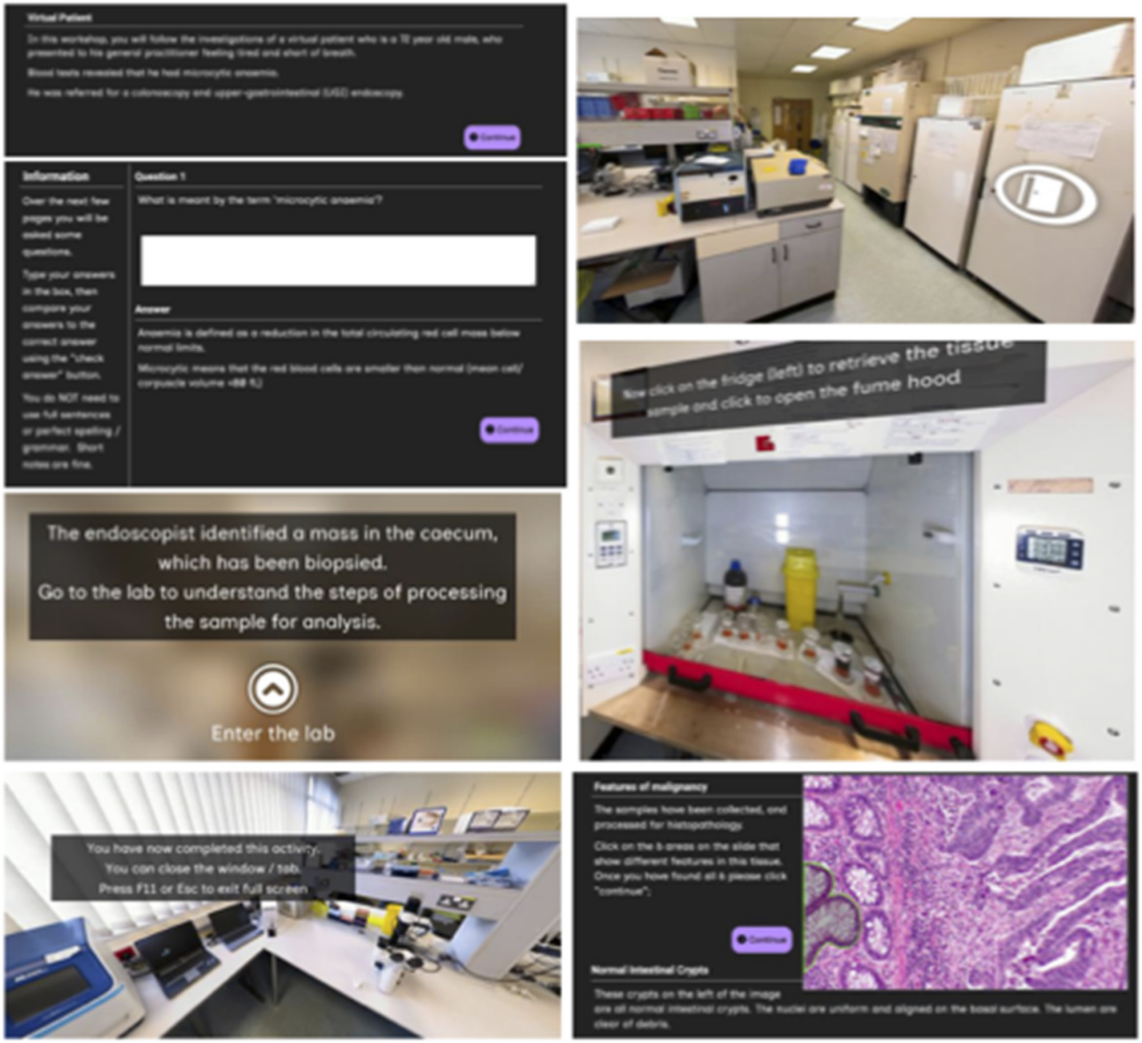
Representative screenshots of the developed VL-Lab to illustrate features incorporated into the design.

A conventional workbook was previously used to deliver histopathology teaching content using a flipped classroom approach and Socratic questioning techniques to promote active learning. This workbook formed the basis for the development of the VR-Lab, which further expanded the narrative approach using a patient journey to include sample processing and the role of a multi-disciplinary team in the decision-making process (Figure 1). Students were provided opportunities to answer questions related to the core content, prior to the provision of model answers (Figure 1).

Developing an interactive resource requires the content to be presented differently from other methods, such as a workbook in a word-processor format. Consequently, to ensure a seamless experience, the VR-Lab was created in collaboration with the Digital Education Team in the Cardiff Learning and Teaching Academy; an internal University department focused on enhancing education and the student experience.

The VR-Lab was developed as a lightweight, web-based, Desktop-VR experience due to the self-directed nature of the activity and the scale of deployment, in line with an internal framework for the development of immersive learning activities (Figure 1). This approach maximised the accessibility of the VR-Lab as students only needed a web browser and internet connection to engage with the content.

Technology-wise the VR-Lab utilised a combination of 360-degree photos in 3dVista for the immersive environment and XERTE for the interactivity, both of which are no-code tools. This allowed direct updates to the content to be made during the development process. The 360-degree photos used in the VR-Lab were captured specifically for this purpose at a Cardiff University molecular biology research laboratory (Figure 1).

The workbook content was subsequently updated to reflect the VR-Lab content.

### Resource Delivery

Using Blackboard Ultra, the entire second-year cohort of the Bachelor of Medicine (MBBCh) programme (2023/24-286 medical students, 2024/25-338 medical students) was provided with links to access the VR-Lab, the Microsoft Word workbook, and a link to an anonymous Microsoft Forms evaluation survey. The Blackboard Ultra notification made it clear that a flipped classroom approach was being used, and while students could choose which format they engaged with, the work needed to be completed prior to an in-person session, 10 days later. The anonymous evaluation survey was also used to capture student questions and identify areas where students had difficulty understanding the content. The identified topics, along with an overview of the main learning objectives, were covered in a didactic in-person session.

### Resource Evaluation

Microsoft Forms was used to capture anonymous feedback on the student experience of using the independent learning resources. The results of this feedback were analysed using Microsoft excel. Open-ended questions were used to capture any difficulties (both technical and relating to understanding of the content) as well as general feedback of the resources. Likert scale questions, using a 5-point scale, were used to capture students’ opinions on the content delivery modalities for user-friendliness, engagement levels, ability to aid learning, and clarity (1 = strongly disagree, 2 = disagree, 3 = neutral, 4 = agree, 5 = strongly agree). Microsoft excel was used to undertake descriptive analysis of the outputs, to determine the median and interquartile range (IQR), and to generate visual representation of the results. The link to the evaluation survey was made available to all students using Blackboard Ultra, as well as being embedded in the first and last pages of the VR-Lab and workbook. The authors acknowledge the low response rate to the feedback survey, presumably due to the voluntary nature of participation, and accept this can limit the generalisation of our findings. However, while we considered making the survey compulsory to increase the response rate, we did not wish to compromise the authenticity of the feedback.

#### Ethics and Consent

Not applicable. As this project was classified as a service evaluation, formal research ethics approval was not required, in accordance with guidance provided by the School of Medicine Research Committee at Cardiff University. Consent was not formally recorded, as completion of the feedback survey was entirely voluntary and anonymous; furthermore, no direct quotes from participant responses have been included in this manuscript.

## Results

### Students Preferred to Use the VR-Lab Compared to the Workbook

The VR-Lab and workbook were made available to the 2023/24 and the 2024/25 cohorts of second-year medical students 624 students across the 2 years. Overall, 514 students (82.4%) engaged with the provided resources, as assessed using the student progress information recorded on Blackboard Ultra. Students were given allocated, timetabled time to complete the work 1 week before an in-person Q&A session. 76 (12.2% of the available cohorts) completed the resource evaluation questionnaire within 4 weeks of the teaching delivery. We acknowledge that this low response rate for feedback is a limitation and has a potential to introduce bias into these findings, although the feedback obtained does offer valuable insights into user experience and areas for improvement, providing a useful foundation for future development and broader implementation.

**Figure 2.**
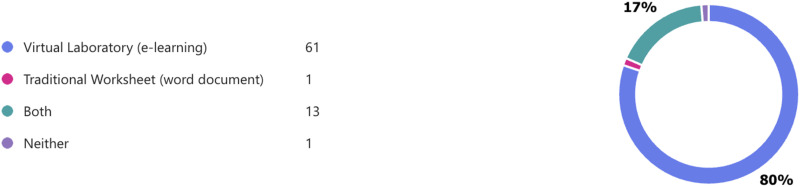
Engagement choice with resource content as indicated by those who completed the feedback survey.

Examination of the data from those who submitted the feedback showed 61 students (80% of respondents) chose to only use the VR-Lab (Figure 2). In contrast, only 1 student chose to exclusively use the workbook (Figure 2). The evaluation data also shows that 13 respondents (17%) chose to use the workbook in conjunction with the VR-Lab resource. Free text comments demonstrated that those who used the workbook did so as a means of notetaking and liked its simplicity. However, all respondents noted the e-learning resource was more engaging, enjoyable and interactive.

### Students Rated the VR-Lab as More Engaging Compared to the Workbook

**Figure 3.**
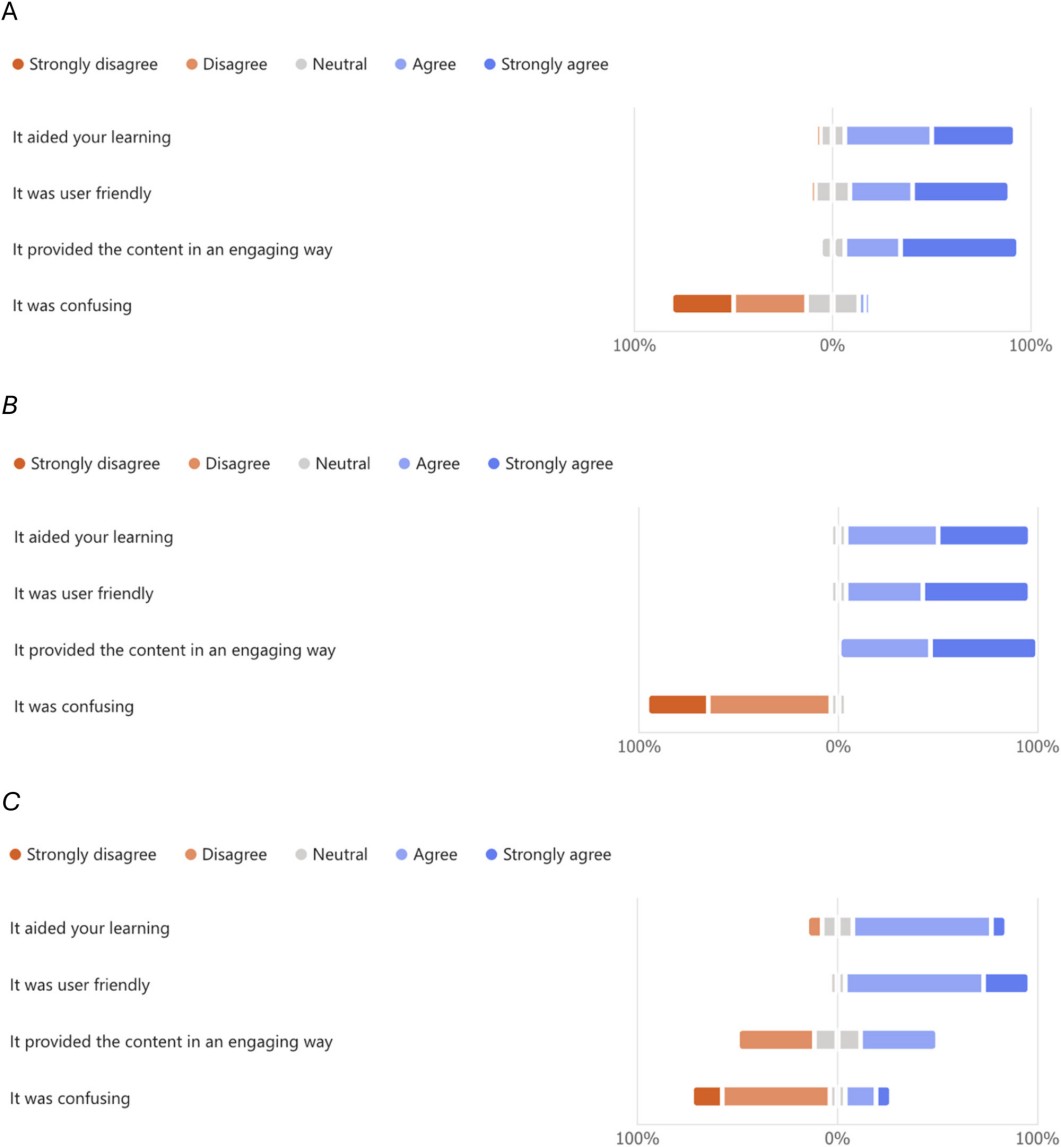
Likert scale rating of the content delivery: A) Rating of VR-Lab by respondents who only used the VR-Lab, B) rating of the VR-Lab by respondents who used both the VR-Lab and workbook (both), c) rating of the workbook by respondents who used both the VR-Lab and workbook. Note the proportions indicated relate to the proportions of those who completed the survey. VR, virtual reality.

Likert scales were used to rate the user-friendliness, engagement levels, ability to aid learning and clarity of the two modes of delivering the learning content (VR-Lab and workbook). The responses obtained overwhelmingly demonstrate that the VR-Lab resource was rated highly as user-friendly (median score 4 (agree), IQR 1), engaging (median score 5 (strongly agree), IQR 1) and perceived to aid learning (median score 4 (agree), IQR 1), while not being confusing (median score 2, IQR 2) (Figure 3A, 3B, Table 1). Likewise, the workbook was rated highly as user-friendly (median score, 4 (agree), IQR 1) and perceived to aid learning (median score, 4 (agree), IQR 0) but compared to the VR-Lab resource, it was not seen as being as engaging (median score 3 (neutral), IQR 2) (Figure 3C, Table 1). Within the free text responses, 23 responses (30.3%) highlighted the provision of model answers with the VR-Lab resource as a feature that was clearly valued. Students noted the instant feedback provided clarity and aided learning. Many student responses noted that the structure and flow of the activity aided engagement with the learning materials, and that the patient narrative supported students putting the learning into context.

**Table 1.** Summary of responses obtained from student feedback.

	It Aided Your Learning	It was User-Friendly	It Provided Content in an Engaging Way	It was Confusing
VR-lab score from VR-lab only responses	median 4, IQR 1	median 4, IQR 1	median 5, IQR 1	median 2, IQR 2
VR-lab score from respondents who used both	median 4, IQR 1	median 5, IQR 1	median 5, IQR 1	median 2, IQR 1
Workbook score from respondents who used both	median 4, IQR 0	median 4, IQR 1	median 3, IQR 2	median 2, IQR 1,
Workbook score from single respondent who used only the workbook	3	4	3	4

VR, virtual reality; IQR, interquartile range.

### Areas for Improvement and Next Steps

The free text comments provided a clear steer towards features that could be used to further improve the VR-Lab and optimise the learning experience. Six respondents (15.8%) asked for a ‘go back’ and/or ‘save progress’ options for easier navigation between sections and to allow recapping of content without having to start over. Some mentioned that when the internet connection dropped, they found it ‘annoying’ and ‘frustrating’ to have to go back to the start rather than skipping sections already completed. This is a feature that should be incorporated and available for future resources of this nature.

The inclusion of video explanations was also a common request from the 23/24 cohort, with one student arguing this would negate the need for the in-person follow-up session. Clearer communication about the provision of such explanations during the in-person Q&A session was provided to the 24/25 cohort, and no comments requesting video explanations were included in the ‘potential improvements’ free text.

Finally, it is worth noting that overall, student feedback indicated very high satisfaction rates and encouragement to develop more resources akin to the VR-Lab. Students noted that this mode of delivery provided very clear content in a highly interactive way. 9 respondents (11.8%) used the free text ‘any other comments’ section to offer positive statements of encouragement.

Therefore, going forward, based on this student feedback, future iterations of the VR-Lab will incorporate key improvements to enhance accessibility and user experience, including the implementation of a ‘go back’ and ‘save progress’ feature to support flexible navigation and reduce frustration caused by connectivity issues. In line with Universal Design for Learning (UDL) principles, we will conduct explicit accessibility checks, such as ensuring keyboard-only navigation, to improve usability and inclusivity, while also strengthening the pedagogical impact of the resource.

## Discussion

Here we have described the creation of a VR-Lab to support genetics and histopathology teaching for second-year medical students and reviewed the preliminary student feedback.

The feedback analysis demonstrates that we successfully met our aim of delivering teaching in an engaging way through the VR-Lab, and this has promoted students’ perception of learning. Our experience developing this VR-Lab is in accordance with the published literature that has proven VR technologies to be effective teaching tools.^9–12^ However, the authors acknowledge that actual student learning was not assessed here and recognise that the absence of objective measures of learning outcomes limits the strength of our conclusions. Future mixed-method approaches that explore ways to assess educational impact more robustly, including performance metrics and longitudinal follow-up is required to address this shortcoming.

Student satisfaction of the VR-Lab and workbook was high, and while many students only used the e-learning resource, the provision of a complementary workbook was beneficial for some. This is an important consideration for future planning of the integration of technology-enhanced learning approaches. Acknowledging the principles of UDL to ensure multiple means of engagement, representation, and expression, educators should utilise a variety of strategies when presenting information.^19^ Our student feedback highlighted the complimentary benefits of providing both ‘new school’ high-tech delivery methods with ‘old school’ traditional workbooks. Indeed, such integration was advocated for in a review by Tawafak et al, (2018),^20^ who emphasised the need for more technology integration throughout different phases of the learning process. Interactive technologies in teaching and learning help students gain greater control over their learning. Conversely, workbooks whether in electronic or paper form, serve as valuable scaffolding resources and offer structured exercises, prompts, and step-by-step guidance, which can anchor students’ self-directed learning. Workbooks provide a tangible framework for practice, reinforcing key concepts and helping learners consolidate their understanding through active learning.^21^

In addition to providing an engaging learning tool, the VR-Lab was initially conceived to provide an alternative hands-on virtual experience, allowing students to have practical exposure to genomic techniques. This experience allows students to develop an understanding of sample processing and handling, DNA extraction, among other parts of histopathology testing. The authors acknowledge that this aim has not been explored in this evaluation, although one student did comment that they would like to have a ‘real lab’ experience. However, further modification of the VR-Lab content could allow students to appreciate the impact of fresh tissue and sample handling on sequencing quality and clinical utility of genomics. This is a potential focus for further iterations of this resource.

The nature of the VR-Lab allows for buildability of content, stations and questions. There is potential for this resource to be further developed to enable students to delve into distinct or specialised areas. For example, it is possible to build it to allow students to appreciate how specific mutations lead to distinct histological features in cancer, thereby showing how genomic data influences diagnosis, treatment decisions, or risk assessments. Modifying the clinical scenario can strengthen the clinical relevance by relating the genomic findings to clinical outcomes, allowing students to explore how targeted therapies (eg, tyrosine kinase inhibitors) are guided by genomic profiles. Some of this content is currently delivered through a didactic lecture and, while it could be part of the VR-Lab and help enhance the students’ knowledge, the authors are cognisant not to over-extend the amount and length of the VR-Lab resource, as student feedback indicated the current resource had a ‘perfect length’.

Another limitation of the current resource is a lack of material that addresses learning relating to the ethical dilemmas related to genetic testing, privacy, and genetic counselling, topics that add further layers of complexity within clinical practice.^22^ This content is also currently delivered in a didactic lecture, but the authors can see the potential for this to be supplemented by alternative technology-based interactive methods.

Overall, preliminary evaluation of this VR-Lab resource highlights the student appetite for, and the benefits of, integrating VR technologies to promote greater interactivity with the learning content.

## References

[1] Unger M Kather JN . Deep learning in cancer genomics and histopathology. Genome Med. 2024;16(1):44. doi:10.1186/s13073-024-01315-6 38539231 PMC10976780

[2] Liu Z Duan T Zhang Y , et al. Radiogenomics: a key component of precision cancer medicine. Br J Cancer. 2023;129(5):741-753. doi:10.1038/s41416-023-02317-8 37414827 PMC10449908

[3] Wang RC Wang Z . Precision medicine: disease subtyping and tailored treatment. Cancers (Basel). 2023;15(15):3837. doi:10.3390/cancers15153837 37568653 PMC10417651

[4] Collins FS Doudna JA Lander ES Rotimi CN . Human molecular genetics and genomics - important advances and exciting possibilities. N Engl J Med. 2021;384(1):1-4. doi:10.1056/NEJMp2030694 33393745

[5] Genome UK: the future of healthcare - GOV.UK. Accessed August 1, 2024. https://www.gov.uk/government/publications/genome-uk-the-future-of-healthcare/genome-uk-the-future-of-healthcare

[6] Mazzoli Smith L Villar F Wendel S . Narrative-based learning for person-centred healthcare: the caring stories learning framework. Med Humanit. 2023;49(4):583-592. doi:10.1136/medhum-2022-012530 37208190 PMC10803961

[7] Milota MM van Thiel GJMW van Delden JJM . Narrative medicine as a medical education tool: a systematic review. Med Teach. 2019;41(7):802-810. doi:10.1080/0142159X.2019.1584274 30983460

[8] McLean. Case-based learning and its application in medical and health-care fields: a review of worldwide literature. J Med Educ Curricular Dev. 2016;3(3):JMECD.S20377. doi:10.4137/JMECD.S20377 PMC5736264 29349306

[9] Elston P Canale GP Ail G Fisher N Mahendran M . Twelve tips for teaching in virtual reality. Med Teach. 2024;46(4):495-499. doi:10.1080/0142159X.2023.2285396 38006603

[10] Masters K Correia R Nemethy K Benjamin J Carver T MacNeill H . Online learning in health professions education. Part 2: tools and practical application: AMEE guide No. 163. Med Teach. 2024;46(1):18-33. doi:10.1080/0142159X.2023.2259069 37740948

[11] Hand C Olaiya R Elmasry M . Virtual reality for teaching clinical skills in medical education. In: Daniela L , ed. New Perspectives on Virtual and Augmented Reality: Finding New Ways to Teach in a Transformed Learning Environment. Routledge; 2020:203-210. doi:10.4324/9781003001874-13

[12] Waugh S Devin J Lam AKY Gopalan V . FE-learning and the virtual transformation of histopathology teaching during COVID-19: its impact on student learning experience and outcome. BMC Med Educ. 2022;22(1):22. doi:10.1186/s12909-021-03066-z 34996435 PMC8740866

[13] Laranjeira M Teixeira MO . Relationships between engagement, achievement and well-being: validation of the engagement in higher education scale. Stud in Higher Educ. 2024;12(1):1–15. doi:10.1080/03075079.2024.2354903

[14] Schnitzler K Holzberger D Seidel T . All better than being disengaged: Student engagement patterns and their relations to academic self-concept and achievement. Eur J Psychol Educ. 2021;36(3):627–652. doi:10.1007/s10212-020-00500-6

[15] Bowden JLH Tickle L Naumann K . The four pillars of tertiary student engagement and success: a holistic measurement approach. Stud in Higher Educ. 2019;46(6):1207–1224. doi:10.1080/03075079.2019.1672647

[16] “The “Classroom Flip”: Using Web Course Management Tools to Become the guide by the side” by J. Wesley Baker. Accessed June 21, 2024. https://digitalcommons.cedarville.edu/media_and_applied_communications_publications/15

[17] Bergmann J Sams A. Flip Your Classroom: Reaching Every Student in Every Class Every Day. 1st ed. International Society for Technology in Education (ISTE); 2012:122.

[18] Kirkwood A Price L . Technology-enhanced learning and teaching in higher education: what is ‘enhanced’ and how do we know? A critical literature review. Learn Media Technol. 2014;39(1):6-36. doi:10.1080/17439884.2013.770404

[19] Luke K . Twelve tips for designing an inclusive curriculum in medical education using Universal Design for Learning (UDL) principles [version 1]. MedEdPublish. 2021;10(1):118. doi:10.15694/mep.2021.000118.1 38486517 PMC10939519

[20] Tawafak RM Romli AB Arshah R bin A Almaroof RAS . Assessing the impact of technology learning and assessment method on academic performance: review paper. EURASIA J Math, Sci & Tech Ed. 2018;14(6):2241–2254. doi:10.29333/ejmste/87117

[21] Graffam B . Active learning in medical education: strategies for beginning implementation. Med Teach. 2007;29(1):38-42. doi:10.1080/01421590601176398 17538832

[22] Berliner J , ed. Ethical Dilemmas in Genetics and Genetic Counseling: Principles through Case Scenarios. Oxford University Press; 2014. doi:10.1093/med/9780199944897.001.0001

